# JN.1: enhanced immune evasion ability propels it to become the predominant strain in China, unlikely to trigger pandemic similar to late 2022

**DOI:** 10.3389/fpubh.2024.1442291

**Published:** 2024-09-26

**Authors:** Peng Du, Meiyi Li, Guohui Wei, Chengbin Guo, Ning Li

**Affiliations:** ^1^Guangzhou Women and Children’s Medical Center, Guangzhou Medical University, Guangzhou, China; ^2^Faculty of Medicine, Macau University of Science and Technology, Macao SAR, China; ^3^Guangzhou National Laboratory, Guangzhou, China

**Keywords:** SARS-CoV-2, JN.1, neutralization, immune evasion, China

## Abstract

**Introduction:**

Due to the widespread presence of susceptible populations, the pandemic caused by BA.5 subbranches swiftly disseminated China, impacting the majority of individuals within a span of 1 to 2 months. Subsequently, XBB and its subbranches became the dominant variants in China.

**Methods:**

We tracked the immune landscape in the population after the SARS-CoV-2 pandemic in late 2022 in China.

**Results:**

Our findings suggested that low levels of neutralizing antibodies against BA.5 subbranches before the pandemic might have contributed to the national outbreak at the end of 2022. The widespread breakthrough infections subsequently increased immunity to BA.5, XBB.1.5/1.9.1, and JN.1, inhibiting a new wave of large-scale infections caused by XBB subbranches in China. Additionally, JN.1 demonstrated enhanced immune evasion capabilities; however, Chinese residents had comparable levels of neutralizing antibodies against JN.1 as those observed for XBB.1.5 among confirmed cases at the end of 2022.

**Discussion:**

We anticipate that JN.1 will replace XBB subbranches as the predominant epidemic variant in subsequent transmissions within China. However, it is unlikely to cause a large-scale spread comparable to that witnessed at the end of 2022, with transmission patterns potentially resembling those observed for XBB post-pandemic.

## Introduction

By the end of 2022, China had implemented a “dynamic zero-COVID” policy for nearly 3 years since the outbreak of COVID-19, resulting in limited incidence of infections among the Chinese population during this period (1). In 2022, Omicron replaced Delta as the most prevalent SARS-CoV-2 variant globally (2). Considering China’s high vaccination coverage and changes in the virus’s transmissibility and pathogenicity, the Chinese government gradually adjusted COVID-19 control measures at the end of 2022. These adjustments included home quarantine or isolation for individuals with mild or no symptoms and the termination of large-scale regional-wide testing (3). Due to the widespread presence of susceptible populations, the pandemic caused by BA.5 subbranches swiftly disseminated nationwide, impacting the majority of individuals within a span of 1 to 2 months. Subsequently, XBB and its subbranches became the dominant variants in China.

The BA.2.86 variant was first discovered in August 2023, and differs from the XBB lineage circulating in China in phylogenetic development. Compared to XBB and BA.2, BA.2.86 has over 30 mutations in the spike protein. However, multiple studies have indicated that its immune evasion capability is comparable to well-known XBB variants like XBB.1.5 and EG.5.1 (4–9). Despite lacking evident growth advantages during transmission, its limited population spread provides an opportunity for the accumulation of immune escape mutations (10).

The JN.1 variant a descendant of BA.2.86, was first identified on August 25, 2023, and designated as a variant of interest (VOI) on December 18, 2023. JN.1 has superseded XBB as the predominant epidemic variant in numerous countries worldwide (10), yet little is known about its immune evasion potential across diverse immunological backgrounds.

China’s distinctive model for epidemic prevention and control, along with its vaccination strategy, makes it challenging to draw conclusions from the epidemic development patterns observed in other countries or regions. Consequently, it is imperative to conduct long-term monitoring of the population following a major outbreak to comprehend the immunization status and subsequently adjust the immunization strategy promptly.

Neutralizing antibodies play a pivotal role in protecting the human body by binding to viral surface proteins and impeding their interaction with host cell receptors (11). The titers of neutralizing antibodies hold significant predictive value for conferring immune protection against SARS-CoV-2 infection (12). Simultaneously, population-level assessment of neutralizing antibodies is essential for evaluating herd immunity and guiding public health responses (13). In our previous studies, we have established a mature system to investigate the neutralization capacity of serum against SARS-CoV-2 under diverse immune backgrounds and assess the immune evasion potential of variants using pseudovirus neutralization experiments (14–17). This study longitudinally tracked the population in Guangdong Province since the onset of the pandemic (December 2022), examined the neutralization efficacy of serum from different populations at various time points, and investigated JN.1’s ability to evade immunity within the Chinese context. These findings could provide valuable insights for formulating epidemic prevention and control policies in China.

## Methods

### Cell culture

The HEK293T (CL-0005; Procell) and 293 T-ACE2-TMPRSS22 (CL0015; VectorBuilder) cell lines were cultured in Dulbecco’s Modified Eagle Medium (DMEM) (C11995500BT; Gibco) supplemented with 10% FBS (10099141C; Gibco) and 1% penicillin–streptomycin (15140122; Gibco). The cells were maintained at 37°C in a 5% CO2 incubator and subcultured every 3–4 days using 0.25% trypsin (25200072; Gibco).

### Preparation and quantification of pseudoviruses

The spike gene sequences of BA.5, XBB.1.5/1.9.1 and JN.1 were synthesized or generated through site-directed mutagenesis and subsequently cloned into the pcDNA3.1(+) vector. The High Fidelity DNA Polymerase Mix (P525; Vazyme) and Exnase II (C214; Vazyme) were used for the polymerase chain reaction (PCR) mix and recombinase enzyme. The preparation and quantitative analysis methods for pseudoviruses were established in our previous article (14). Specifically, the EasyPure HiPure Plasmid MaxiPrep Kit (EM121; TransGen Biotech) was used to purify the pcDNA3.1(+)-spike, pLOVE-luciferase-EGFP, and psPAX2 plasmids amplified by *Escherichia coli*. Using Lipofectamine 3,000 (L30000015; Invitrogen), 12 μg of pLOVE-luciferase-EGFP plasmid, 6 μg of psPAX2 plasmid, and 2 μg of pcDNA3.1(+)-spike plasmid were co-transfected into 8 × 10^6,293 T cells in 100 mm cell culture dishes. After 6–8 h of transfection, the medium was replaced with 10 mL of fresh medium. Supernatant containing pseudoviruses was collected 48 h post-transfection, centrifuged at 4°C for 10 min at 200 × g, and filtered through a 0.45 μm membrane filter. RNA extraction from the pseudoviruses was performed using the TaKaRa Minibest Viral RNA/DNA Extraction Kit Ver.5.0 (9,766; TaKaRa). Reverse transcription was carried out with the HiScript III All-in-One RT SuperMix Perfect for qPCR kit (R333-01; Vazyme). The titer of pseudoviruses was determined using the TransLvTM Lentivirus qPCR Titration Kit (FV201; TransGen Biotech). The specificity of the pseudoviruses was tested using human serum samples from individuals not infected with SARS-CoV-2 and mouse serum samples from those vaccinated with the XBB.1.5/1.9.1 spike trimer protein (Supplementary Figure S1).

### Human sera

All serum samples were obtained via intravenous blood collection, isolated by centrifugation at 4000 rpm at 4°C for 10 min, inactivated at 56°C for 30 min, and stored at −80°C.

### Pseudovirus neutralization assay

The use of immune serum in this study was approved by the Ethics Committee of Macao University of Science and Technology. The 293 T-ACE2-TMPRSS2 cells (1 × 10^4^/well) were seeded in a 96-well white cell culture plate and incubated for 12 h prior to the experiment. The human sera were diluted with DMEM containing 10% FBS in a round-bottom cell culture plate, followed by pre-incubation with SARS-CoV-2 pseudovirus (2 × 10^4^ RLU) at 37°C for 1 h. Each plate also included six virus control wells (without sera) and six control wells (without virus and sera). After incubation, the mixture was transferred to a white plate and further incubated at 37°C for an additional 12 h before replacing the medium. After 48 h, luciferase substrate (11404ES80; Yeasen) was added to each well and the fluorescence signal was measured using LumiStation 1800 (Shanghai Flash Biotechnology Co., Ltd). The median effective dose (ED_50_) of the sample was calculated using the Reed-Muench method (18).

### Quantification and statistical analysis

The software GraphPad Prism 8.0.1 was used for data visualization and statistical analysis. Results were presented as median with interquartile range (IQR) or geometric mean titer (GMT). The Mann–Whitney test was utilized with a significance level set at *p* = 0.05 (*p < 0.05, **p < 0.01, ***p < 0.005, ****p < 0.001).

## Results

### Population data

In this study, five groups of serum samples were collected at different time points following the large-scale outbreak of SARS-CoV-2 infection in China at the end of 2022. These samples were used to investigate the neutralizing activity against several SARS-CoV-2 variants, particularly the currently circulating JN.1. The first group consisted of serum samples from 99 volunteers (median age: 28 years, interquartile range (IQR): 25–33 years, 35.4% male) who received a three-dose vaccination of an inactivated virus vaccine prior to the large-scale outbreak in Zhuhai, Guangdong Province. The second dose was administered on day 28 after the first dose, and the third dose on day 256 after the second dose. Serum samples were collected on day 14 after the third dose. These samples provided insight into the immune background of the population before the outbreak, given that the majority of the Chinese population received inactivated virus vaccines.

The second group included 36 diagnosed patients (median age: 30 years, IQR: 27–34 years, 33.3% male) recruited in Guangzhou, Guangdong Province during the first month following the outbreak (December 2022). Serum samples were collected on day 29 after diagnosis. Among these, 31 individuals (median age: 30 years, IQR: 27–35 years, 32.3% male) were infected for the first time after receiving a three-dose inactivated virus vaccine, while five individuals (median age: 30 years, IQR: 28–33 years, 40.0% male) were infected for the first time after receiving other vaccination schemes. These samples were used to study the impact of infection on the population’s immune status.

According to the China CDC, the large-scale infections had essentially ended by the end of February 2023. To understand the immune status of the population after the large-scale infection and the differences among various age groups, samples were collected in March 2023 from people undergoing health check-ups at Zhuhai People’s Hospital. This group consisted of 90 individuals (median age: 35 years, IQR: 5–64 years, 55.6% male): 30 minors (median age: 3 years, IQR: 2–5 years, 55.6% male) under the age of 15, 30 adults (median age: 35 years, IQR: 32–40 years, 66.7% male) aged 16–60, and 30 older adults (median age: 67 years, IQR: 64–71 years, 53.0% male) aged over 60.

Starting from April 2024, XBB and its sub-lineages gradually became the dominant strains in China, remaining the principal circulating variants until December 2023. JN.1 was first detected on August 25, 2023, and by December 2023, it had replaced XBB as the predominant strain in multiple countries worldwide. To understand the population’s immunity against these two major circulating strains, we collected the most recent samples (October 2023 and January 2024) for analysis. Given that SARS-CoV-2 infections can lead to more severe outcomes in older adults, we specifically focused on collecting samples from individuals aged 60 and above in the fourth group. The fourth group included 100 volunteers (median age: 59 years, IQR: 54–68 years, 17.0% male) randomly recruited from Zhuhai communities in the eleventh month after the outbreak. Among these, 54 adults (median age: 55 years, IQR: 49–57 years, 13.0% male) were below 60 years old, and 46 older adults (median age: 69 years, IQR: 65–74 years, 21.7% male) were aged 60 and above. The fifth group consisted of 114 adult volunteers (median age: 33 years, IQR: 30–35 years, 23.7% male) recruited from Guangzhou Women and Children’s Medical Center in January 2024. Demographic information for different groups and detailed information on sample collection time points are shown in Table 1.

**Table 1:** Characteristics of study subjects.

Characteristics	Three-dose inactivated virus vaccine	Diagnosed cases in December 2022	Three-dose inactivated virus vaccine + infected	Population in March 2023	Population in October 2023	Population in January 2024
No. of participants	99	36	31	90	100	114
Age (median, IQR)	28 (25–33)	30 (27–34)	30 (27–35)	35 (5–64)	59 (54–68)	33 (30–35)
Sex (%)						
Male	35 (35.4)	12 (33.3)	10 (32.3)	50 (55.6)	17 (17)	27 (23.7)
Female	64 (64.6)	23 (66.7)	21 (67.7)	40 (44.4)	83 (83)	87 (76.3)
Vaccination interval						
Interval between the first and second vaccinations (median, IQR)	28 (22–34)	NA	32 (28–36)	NA	NA	NA
Interval between the second and third vaccinations (median, IQR)	256 (223–294)	NA	206 (196–277)	NA	NA	NA
Sample collection timing						
Days from third vaccine dose to sampling (median, IQR)	14 (14–14)	NA	386 (304–427)	NA	NA	NA
Days from diagnosis to sampling (median, IQR)	NA	29 (26–32)	29 (26–33)	NA	NA	NA

IQR, interquartile range; NA, not available.

### Neutralizing activity of serum against BA.5, XBB.1.5/1.9.1 and JN.1 in diagnosed population in December 2022

**Figure 1:**
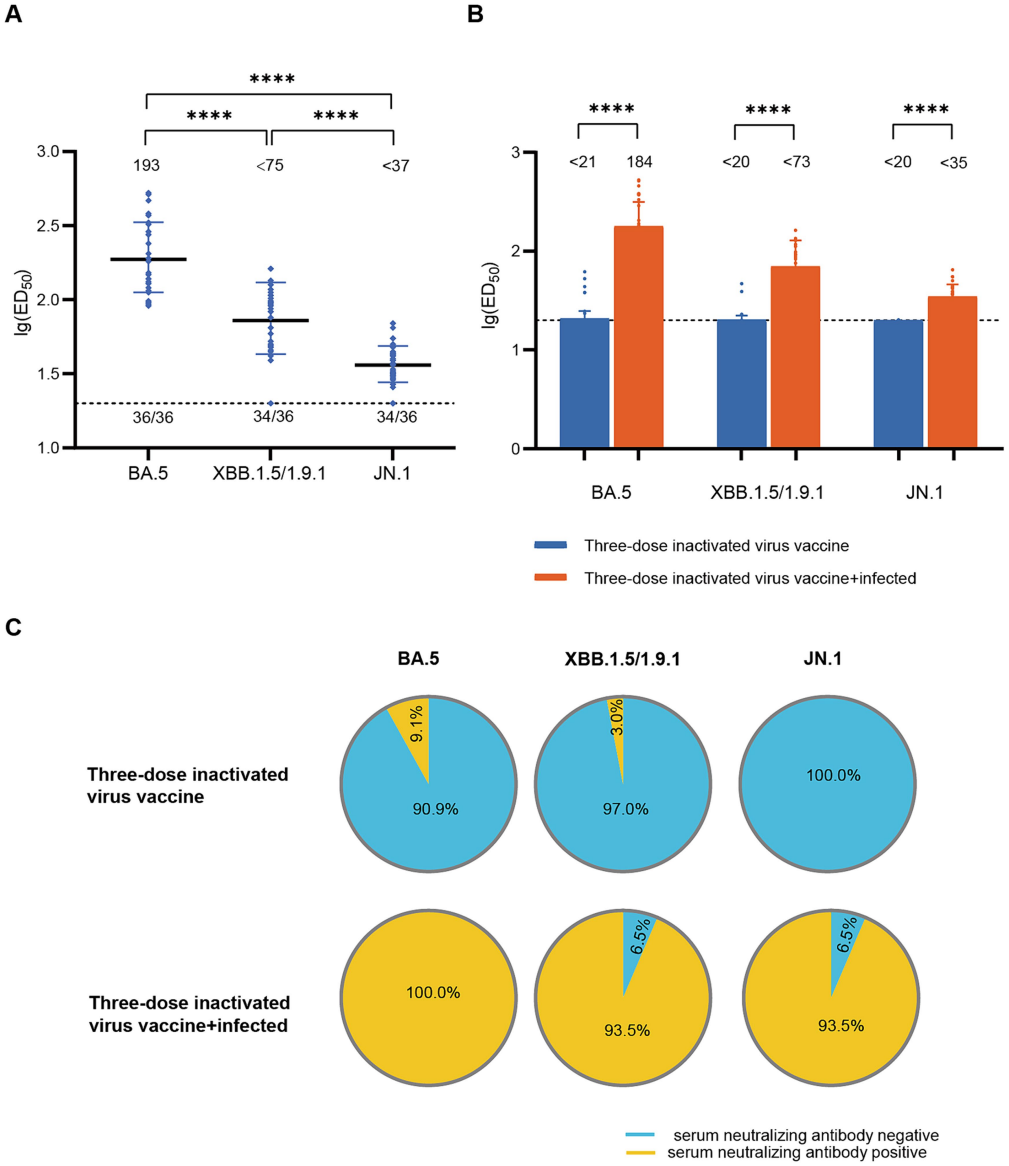
Neutralizing activity of sera in the diagnosed population in December 2022. **(A)** Neutralizing activity against BA.5, XBB.1.5/1.9.1 and JN.1 in the diagnosed population in December 2022 (*n* = 36). **(B)** Comparison of serum neutralizing antibody titers between individuals infected after receiving three doses of the inactivated virus vaccine (*n* = 31) and those who received only three doses of the inactivated virus vaccine (*n* = 99). The values below the line represent the GMT of ED_50_ for each dataset. “*” Indicates a significant statistical difference. The dashed lines represent the limit of detection. In the statistical analysis, an ED_50_ lower than a 20-fold dilution was denoted as 20 for these samples. The values below the dashed line indicate the proportion of positive samples out of the total samples (number of positive samples/total number of samples). ED50, median effective dose, represents the serum dilution required to inhibit 50% of viral infection. **(C)** Comparison of serum neutralizing antibody positive rates between individuals infected after receiving three doses of the inactivated virus vaccine (*n* = 31) and those who received only three doses of the inactivated virus vaccine (*n* = 99). The top section of the image represents different viral variants. The pie charts above depict the positivity rate of neutralizing antibodies in sera from individuals who received three doses of the inactivated vaccine only. The pie charts below show the positivity rate of neutralizing antibodies in sera from individuals who received three doses of the inactivated vaccine and were subsequently infected. Yellow indicates positive sera, while blue indicates negative sera.

To assess the impact of infection on population immunity, we initially examined the neutralizing antibody titers against BA.5, XBB.1.5/1.9.1, and JN.1 in the sera of 36 recovered patients in December 2022. The results showed robust immune responses against all three variants in all serum samples (Figure 1A). The geometric mean titers (GMT) and positive rates were: BA.5 (193, 100%), XBB.1.5/1.9.1 (<75, 94.4%), JN.1 (<37, 94.4%). Notably, the neutralizing activity against JN.1 was significantly lower compared to BA.5 and XBB.1.5/1.9.1 by approximately 5-fold and 2-fold respectively, indicating a stronger immune evasion capability for the JN.1 variant. Comparing individuals infected after receiving three doses of an inactivated virus vaccine to those who only received the inactivated virus vaccine without subsequent infection, infection substantially enhanced serum neutralizing activity (*p* < 0.001). Post-infection neutralizing activity increased from (<21, <20, <20) to (184, <73, <35) (Figure 1B), while positivity rates rose from (9.1, 3.0, 0.0%) to (100, 93.5, 93.5%) (Figure 1C).

### Neutralizing activity of BA.5 in the population in March 2023

**Figure 2:**
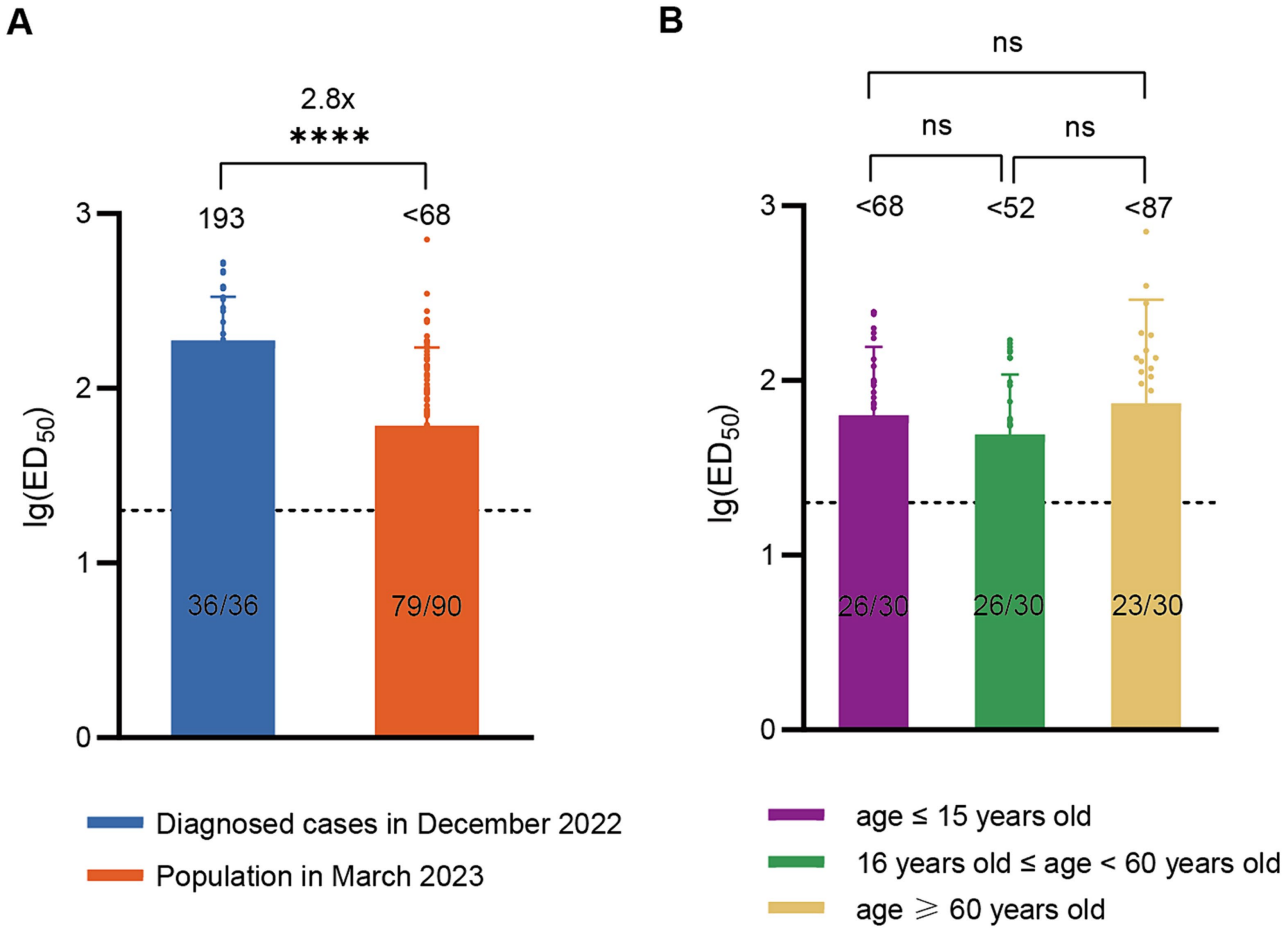
Neutralizing activity of sera against BA.5 in the population in March 2023. **(A)** Comparison of neutralizing antibody titers against BA.5 between the population in March 2023 (*n* = 90) and the diagnosed population in December 2022 (*n* = 36). **(B)** Neutralizing antibody titers against BA.5 in age-stratified groups in March 2023, with 30 individuals per age group. The values below the line represent the GMT of ED_50_ for each dataset. The values above the line represent the fold change. “*” Indicates a significant statistical difference. The dashed lines represent the limit of detection. In the statistical analysis, an ED_50_ lower than a 20-fold dilution was denoted as 20 for these samples. ED_50_: median effective dose. The values below the dashed line indicate the proportion of positive samples (ED_50_ ≥ 20) out of the total samples (number of positive samples/total number of samples).

According to China CDC data, the positive rate of COVID-19 nucleic acid tests in China increased initially and then decreased after December 9, 2022, peaking at 29.2% on December 25 and declining to 1.4% by February 23, 2023 (19), indicating that this wave of COVID-19 epidemic had essentially subsided by the end of February 2023. To assess post-epidemic immunity, serum neutralizing antibody titer tests were conducted on a cohort of 90 volunteers in March 2023. The results revealed a GMT of <68 for neutralizing antibodies against BA.5 with a positivity rate of 83.3%. Compared to diagnosed patients in December 2022, there was a significant decline (approximately 2.8-fold) in serum neutralizing activity against BA.5 in March 2023 (Figure 2A). The levels of neutralizing antibodies may vary among different age groups following infection. To evaluate the immunity status across different age groups after this wave of the pandemic, we conducted further age-stratified analysis on these 90 volunteers. Although the GMT values were slightly higher in older adults (<87) compared to juveniles (<68) and adults (<52), the differences were not statistically significant (Figure 2B).

### Neutralization activities of XBB.1.5/1.9.1 and JN.1 in October 2023 and January 2024

To understand current population immunity to the main variant XBB and the potential epidemic variant JN.1, we recruited 100 volunteers from Zhuhai in October 2023 and 114 volunteers from Guangzhou in January 2024.

**Figure 3:**
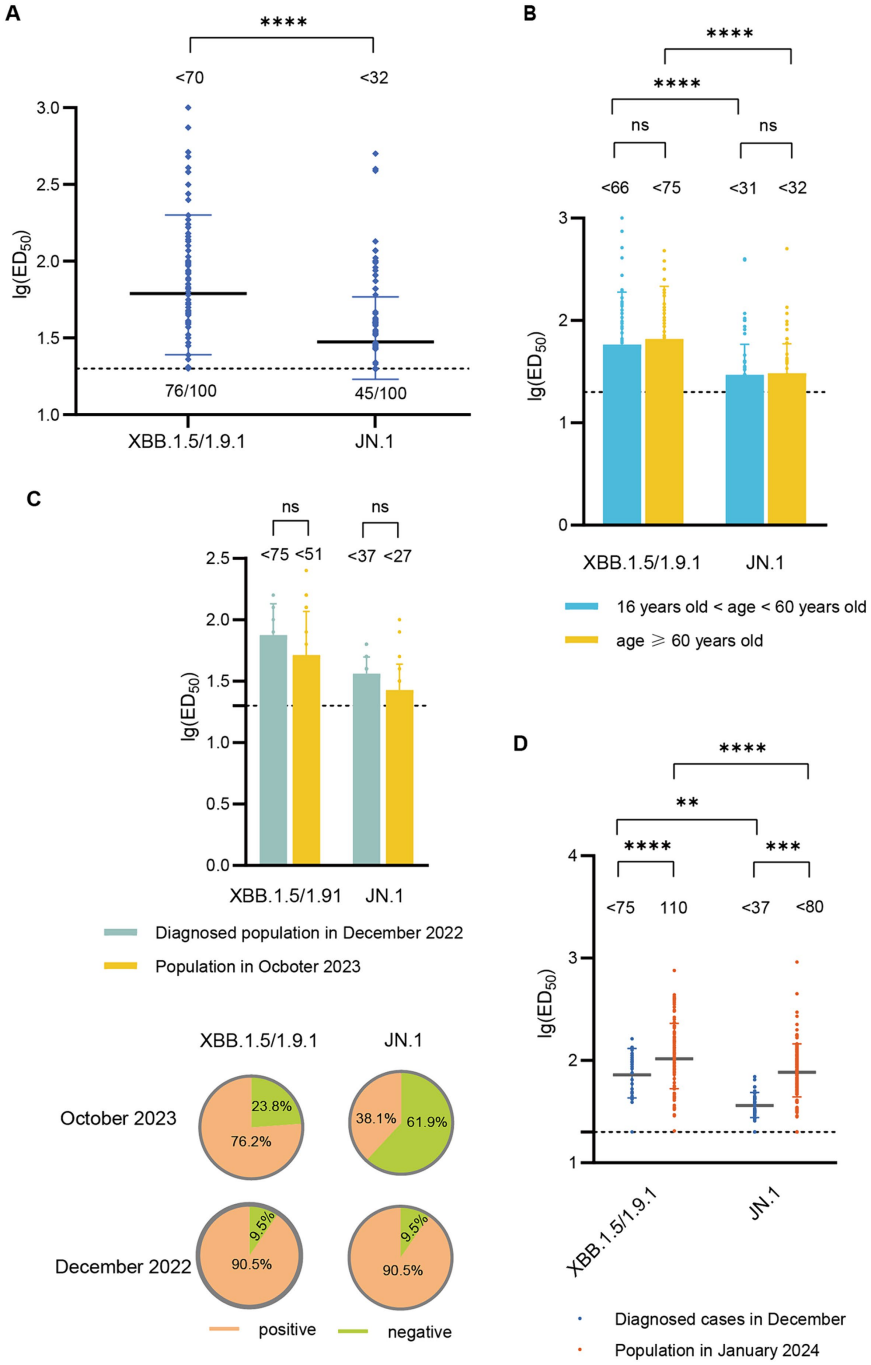
Neutralizing activity of sera against XBB.1.5/1.9.1 and JN.1 in the population in October 2023 and January 2024. **(A)** Neutralizing activity of sera against XBB.1.5/1.9.1 and JN.1 in the population in October 2023 (*n* = 100). **(B)** Comparison of neutralizing activity of sera against XBB.1.5/1.9.1 and JN.1 in the population in October 2023 by age group (16 < age < 60, *n* = 54; age ≥ 60, *n* = 46). **(C)** The results of the age-matched comparison between the population in October 2023 and the diagnosed population in December 2022 (*n* = 21). The bar chart displays the neutralizing antibody titers, while the pie chart shows the seropositivity rates. **(D)** Comparison of neutralizing activity of sera against XBB.1.5/1.9.1 and JN.1 in the population in January 2024 and the diagnosed population in December 2022. The values below the line represent the GMT of ED_50_ for each dataset. “*” Indicates a significant statistical difference. The dashed lines represent the limit of detection. In the statistical analysis, an ED_50_ lower than a 20-fold dilution was denoted as 20 for these samples. The values below the dashed line indicate the proportion of positive samples (ED_50_ ≥ 20) out of the total samples (number of positive samples/total number of samples).

In October 2023, the GMT of neutralizing antibody titers against XBB.1.5/1.9.1 was <70, with a serum positive rate of 76.0%. The GMT for neutralizing antibodies against JN.1 was <32 (2.2 times lower than that of XBB.1.5/1.9.1), with a serum positive rate of 45.0% (Figure 3A). To investigate whether there are differences in neutralizing antibody levels across different age groups, we categorized participants (October 2023) into two age groups (>16- < 60 years old and ≥ 60 years old). We found no significant differences in the neutralizing antibody titers and seropositivity rates against XBB.1.5 and JN.1 between these two age groups (Figure 3B).

Subsequently, we compared the neutralizing antibody titers between individuals in October 2023 and those diagnosed in December 2022. Due to significant differences in the age composition between the two groups, we performed age-matched comparisons, successfully matching 21 pairs. The neutralizing antibody titers against XBB.1.5/1.9.1 and JN.1 in the October 2023 cohort (<52, <27) were lower than those in the December 2022 cohort (<75, <37), though the difference was not statistically significant. Additionally, the seropositivity rates for XBB.1.5/1.9.1 and JN.1 were lower in the October 2023 cohort (76.2, 38.1%) compared to the December 2022 cohort (90.5, 90.5%) (Figure 3C).

In January 2024, the GMT of neutralizing antibody titers against XBB.1.5/1.9.1 was 110, with a 100% positive rate. The GMT for neutralizing antibodies against JN.1 was <80 (1.4 times lower than that of XBB.1.5/1.9.1), but the positive rate remained high at 97.4%. These titers surpassed those detected in diagnosed patients in December 2022 (Figure 3D).

## Discussion

Before December 7, 2023, China’s unique epidemic prevention and control policies effectively protected the majority of the population from exposure to the SARS-CoV-2 virus. The population’s immunity was mainly derived from vaccination (20). However, following the lifting of these measures, a large-scale spread of SARS-CoV-2 occurred, with most people becoming infected within a 1 to 2-month period (21, 22). Since then, COVID-19 has persisted at a relatively low level in China. The country’s distinct epidemic prevention and control mode and vaccination strategy have yielded a unique pattern of epidemic development. As such, long-term monitoring of the population following the large-scale epidemic is essential. This not only helps to understand the population’s immune status and provides a reference for adjusting China’s immunization policies, but also aids in understanding the transmission patterns of emerging infectious diseases under varying prevention and control strategies.

According to data from China CDC, from December 1, 2022, to February 14, 2023, BF.7 and its subbranches were the predominant circulating variants in Beijing, Tianjin, and Inner Mongolia. In Jiangsu Province, BF.7 and its sub-branches were present in proportions roughly equivalent to BA.5.2 and its subbranches. In other provinces, BA.5.2 and its subbranches were the prevailing variants during this epidemic phase (19, 23). Thus, this wave of the epidemic was primarily driven by the BA.5 subbranches. Simultaneously, starting from April 2023, XBB and its subbranches became the main circulating variants in China until December 2023.^1^ Furthermore, a new variant named BA.2.86 was first identified in Israel and Denmark in August 2023. This variant is a descendant of BA.2, with 43 additional mutations, including 34 mutations in the spike protein (9). Since October 2023, there has been a global increase in the prevalence of the BA.2.86 sequence. Although it has not shown a significant transmission advantage, concerns have arisen due to its heightened binding affinity with angiotensin-converting enzyme 2 (ACE2) compared to XBB.1.5/1.9.1 and EG.5 (6). This increased affinity may allow it to tolerate additional substitutions in the spike protein that negatively affect ACE2 binding affinity, making it more potent in escaping neutralization than the original BA.2.86 (6). JN.1, an evolved form of the BA.2.86 variant, was first identified on August 25, 2023. It carries an additional mutation (L455S) in the spike protein, potentially enhancing its immune evasion capability. Currently, JN.1 has replaced XBB as the main epidemic variant in many countries worldwide (10). According to reports from the China CDC, the proportion of JN.1 has shown an upward trend since December 2023 (see text footnote 1). To study the immune status of individuals at different time points after the outbreak and the immune escape ability of JN.1 under China’s immune background, as well as to predict the impact of JN.1 on the epidemic in China, we collected serum samples from people at various time points. We tested the neutralizing activity of these sera against three SARS-CoV-2 variants: BA.5, XBB.1.5/1.9.1, and JN.1.

Our study showed that sera collected 14 days after three doses of inactivated virus vaccine had a neutralizing antibody titer of less than 21 (the detection limit is 20), and the serum positive rate was only 9.1%. Since the SARS-CoV-2 vaccine used by most residents in China was the inactivated virus vaccine, this may represent the highest immune level of the Chinese population to BA.5 and its subbranches before the epidemic outbreak. By the time the epidemic policy was released in December 2022, the last vaccination for the vast majority of residents had been much longer than 14 days prior, and the neutralizing antibody levels produced by the vaccine would have decreased over time (24). Therefore, the serum positive rate for BA.5 in the population before the outbreak may have been lower, potentially contributing to the rapid large-scale infections following the relaxation of prevention and control policies.

Analyzing the serum of diagnosed patients demonstrates that the immune response to BA.5, XBB.1.5/1.9.1, and JN.1 significantly increases following infection. From early February to mid-April, the prevalence of COVID-19 in Chinese influenza-like cases remained low. However, during this period, XBB and its subbranches were widely circulating globally (25, 26). This indicates that the immune barrier established by widespread infection effectively reduced subsequent variant infections within the population. Comparing the serum of the population in March 2023 with that of diagnosed patients in the first month of the large-scale outbreak, it was found that the serum neutralizing antibody titer decreased by 2.8 times, indicating that the immunity induced by mass infection began to decline. This was consistent with the gradual increase in the positive rate of COVID-19 in Chinese influenza-like cases from mid-April.

From mid-April 2023, the proportion of COVID-19 positive cases reported by sentinel hospitals in China gradually increased, peaking at 42.5% in May and subsequently declining to less than 5% by the end of October 2023. Meanwhile, JN.1, a subbranch of BA.2.86, was initially detected on August 25, 2023, and its global incidence has been steadily rising, potentially emerging as the dominant variant. To assess the immunity of the population against XBB and JN.1 following the conclusion of the recent outbreak, we recruited volunteers at the end of October 2023 and performed age-matched comparisons with patients diagnosed in December 2022. The results indicated a decline in both neutralizing antibody titers and seropositivity rates in the October 2023 cohort compared to the December 2022 cohort. This suggests that the protective effect induced by infection diminishes over time, underscoring the importance of timely vaccination to enhance population immunity.

The results from the most recent population sample (January 2024) demonstrated significantly higher neutralizing antibody titers against XBB.1.5/1.9.1 compared to those in diagnosed patients in December 2022. This was consistent with the further decrease in the proportion of COVID-19 positive cases in influenza-like cases at the end of December 2023. These findings indicate that the current population exhibits robust resistance to XBB and its variants.

Regarding JN.1, our analysis revealed its superior immune evasion capability compared to BA.5 and XBB.1.5/1.9.1 across all population groups. This suggests that JN.1 is likely to replace XBB and its variants as the predominant epidemic variant during subsequent transmission in China. It was also found that the neutralizing antibody titers of JN.1 in the population in January 2024 were the highest since the outbreak. Given that the increase in population immunity after the outbreak mainly comes from the breakthrough infection of XBB and its subbranches or administration of XBB-containing vaccines, we speculated that vaccines containing XBB may still be effective against JN.1. Moreover, the neutralizing antibody titer (<80) and serum positive rate (97.4%) of JN.1 in January 2024 were comparable to those recorded for XBB.1.5/1.9.1 among diagnosed cases during the pandemic (neutralizing antibody titer: <75, serum positive rate: 94.4%). Consequently, we hypothesize that JN.1 may not cause a large-scale spread in China similar to that seen in late 2022, and its transmission mode would likely resemble that of XBB and its subbranches within China post-pandemic.

## Conclusion

We studied the immune status of the population from the large-scale outbreak of SARS-CoV-2 in China to the recent period, which is of great significance for understanding the changing patterns of population immunity under different epidemic prevention and control models. Despite the unique immune background of the Chinese population prior to the widespread infections at the end of 2022, similar to observations in other countries, each breakthrough infection significantly enhanced the population’s resistance to SARS-CoV-2. Notably, breakthrough infections increased the breadth of antibody responses, including against potential future variants. This enhancement is crucial for the human adaptation to the continuous evolution of SARS-CoV-2 (27). Additionally, compared with BA.5 and XBB.1.5/1.9.1, JN.1 exhibited a stronger ability for immune escape, which may make it the next major epidemic variant in China. However, the current population’s immunity to JN.1 is similar to that of the population to XBB.1.5/1.9.1 after the large-scale outbreak, suggesting that the transmission mode of JN.1 in China may be similar to that of XBB post-pandemic.

## Limitation

Firstly, the relatively small sample size and the lack of a complete match in demographic characteristics (e.g., age) could lead to deviations in the results. We also lacked vaccination and diagnosis records for individuals other than those diagnosed in December 2022 and those who received three doses of the inactivated virus vaccine. Secondly, the epidemic data in China were obtained from the official website of the China CDC, which lacks original data. Finally, we did not evaluate long-lived plasma cells, memory B cells, and T cell immunity, which could provide more insights into population immunity and epidemic prediction.

## Data Availability

The raw data supporting the conclusions of this article will be made available by the authors, without undue reservation.

## Footnotes

^1^https://www.chinacdc.cn/jkzt/crb/zl/szkb_11803/jszl_13141/202401/t20240110_271902.html
