# Supplementary material for: JN.1: enhanced immune evasion ability propels it to become the predominant strain in China, unlikely to trigger pandemic similar to late 2022
Source: Front Public Health. 2024 Sep 26;12:1442291. doi: 10.3389/fpubh.2024.1442291 (PMC11464334; doi:10.3389/fpubh.2024.1442291)
Supplement: Supplementary file 1 [file Data_Sheet_1.DOC]

**Specificity verification of pseudoviruses**

Neutralization assays were performed using human serum samples from individuals not infected with SARS-CoV-2 and mouse serum samples from those vaccinated with the XBB.1.5/1.9.1 spike trimer protein, following method 4. The results showed that the human serum from individuals not infected with SARS-CoV-2 did not neutralize the pseudoviruses, whereas the mouse serum from those vaccinated with the XBB.1.5/1.9.1 recombinant protein vaccine effectively neutralized the pseudoviruses (Fig.S1).


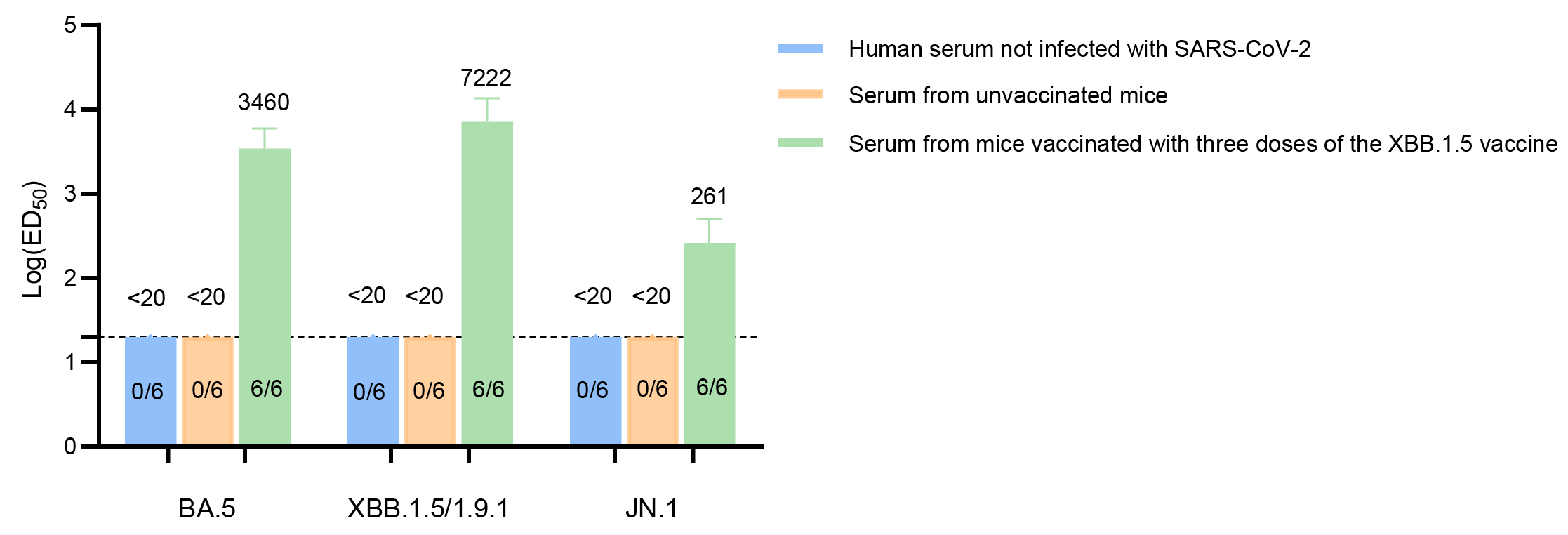


Figure S1 Specificity verification of pseudoviruses

The x-axis represents different pseudoviruses, and the y-axis represents lg(ED_50_). The values presented represent the geometric mean of ED50. The dashed lines represented the limit of detection. In the statistical analysis, an ED_50_ lower than a 20-fold dilution was denoted as 20 for these samples. ED_50_, median effective dose. The values below the dashed line indicate the proportion of positive samples (ED_50_≥20) out of the total samples (number of positive samples/total number of samples). Human serum samples were collected before the discovery of SARS-CoV-2 (n=6). Mouse serum samples were collected from female BALB/c mice that had received three doses of the XBB.1.5/1.9.1 recombinant protein vaccine. 10 μg of XBB.1.5/1.9.1 spike trimer protein (40589-V08H45; Sino Biological) was individually dissolved in 50 μL PBS. Subsequently, 50 μL aluminum hydroxide gel (aluminum content: 10 mg/mL) adjuvant (Alhydrogel adjuvant 2%, vac-alu-250; InvivoGen) was added, and the mixture was vigorously shaken for 5 minutes to prepare vaccines. BALB/c female mice (6-8 weeks old, n=6), purchased from Gempharmatech-GD for immunization, were intramuscularly injected at 0, 1, and 3 weeks. Serum samples were collected by retro-orbital hemorrhage 3 weeks after the last immunization. The mice were fully anesthetized before sample collection. The sera were isolated by centrifugation at a speed of 4000 rpm and maintained at a temperature of -80°C.
